# Correction: Chen, S. *et al.* The Tradeoff Analysis for Remote Sensing Image Fusion Using Expanded Spectral Angle Mapper. 

**DOI:** 10.3390/s120912374

**Published:** 2012-09-11

**Authors:** Shaohui Chen, Hongbo Su, Renhua Zhang, Jing Tian, Lihu Yang

**Affiliations:** Key Laboratory of Water Cycle and Related Land Surface Processes, Institute of Geographic Sciences and Natural Resources Research, Chinese Academy of Sciences, 11A, Datun Road, Chaoyang District, Beijing 100101, China; E-Mails: chenshaohu@gmail.com (S.C.); zhangrh@igsnrr.ac.cn (R.Z.); tianj.04b@igsnrr.ac.cn (J.T.); yanglihu@igsnrr.ac.cn (L.Y.)

There are two mistakes in this article [1]. On page 523, lines 10–11, the sentence “the value of the SAM is 1, but the value of the ESAM is less than 1” should be “the value of the SAM is 0, but the value of the ESAM is great than 0”. Line 12, “but the value of the ESAM is less than 1” should be “but the value of the ESAM is great than 0 for even *n*”.
